# Adverse outcomes after arthroscopic partial meniscectomy: a study of 700 000 procedures in the national Hospital Episode Statistics database for England

**DOI:** 10.1016/S0140-6736(18)31771-9

**Published:** 2018-11-17

**Authors:** Simon G F Abram, Andrew Judge, David J Beard, Andrew J Price

**Affiliations:** a Nuffield Department of Orthopaedics, Rheumatology and Musculoskeletal Sciences, University of Oxford, Oxford, UK; b Musculoskeletal Research Unit, University of Bristol, Bristol, UK

## Abstract

**Background:**

Arthroscopic partial meniscectomy is one of the most common orthopaedic procedures worldwide. Clinical trial evidence published in the past 6 years, however, has raised questions about the effectiveness of the procedure in some patient groups. In view of concerns about potential overuse, we aimed to establish the true risk of serious complications after arthroscopic partial meniscectomy.

**Methods:**

We analysed national Hospital Episode Statistics data for all arthroscopic partial meniscectomies done in England between April 1, 1997, and March 31, 2017. Simultaneous or staged (within 6 months) bilateral cases were excluded. We identified complications occurring in the 90 days after the index procedure. The primary outcome was the occurrence of at least one serious complication within 90 days, which was defined as either myocardial infarction, stroke, pulmonary embolism, infection requiring surgery, fasciotomy, neurovascular injury, or death. Logistic regression modelling was used to identify factors associated with complications and, when possible, risk was compared with general population data.

**Findings:**

During the study period 1 088 782 arthroscopic partial meniscectomies were done, 699 965 of which were eligible for analysis. Within 90 days, serious complications occurred in 2218 (0·317% [95% CI 0·304–0·330]) cases, including 546 pulmonary embolisms (0·078% [95% CI 0·072–0·085]) and 944 infections necessitating further surgery (0·135% [95% CI 0·126–0·144]). Increasing age (adjusted odds ratio [OR] 1·247 per decade [95% CI 1·208–1·288) and modified Charlson comorbidity index (adjusted OR 1·860 per 10 units [95% CI 1·708–2·042]) were associated with an increased risk of serious complications. Female sex was associated with a reduced risk of serious complications (adjusted OR 0·640 [95% CI 0·580–0·705). The risk of mortality fell over time (adjusted OR 0·965 per year [95% CI 0·937–0·994]). Mortality, myocardial infarction, and stroke occurred less frequently in the study cohort than in the general population. The risks of infection and pulmonary embolism did not change during the study, and were significantly higher in the study cohort than in the general population. For every 1390 (95% CI 1272–1532) fewer knee arthroscopies done, one pulmonary embolism could be prevented. For every 749 (95% CI 704–801) fewer procedures done, one native knee joint infection could be prevented.

**Interpretation:**

Overall, the risk associated with undergoing arthroscopic partial meniscectomy was low. However, some rare but serious complications (including pulmonary embolism and infection) are associated with the procedure, and the risks have not fallen with time. In view of uncertainty about the effectiveness of arthroscopic partial meniscectomy, an appreciation of relative risks is crucial for patients and clinicians. Our data provide a basis for decision making and consent.

**Funding:**

UK National Institute for Health Research.

## Introduction

Meniscal tears are common but associated with highly variable knee symptoms, signs, and radiological findings.^1^, ^2^ Many meniscal tears are asymptomatic, and knee symptoms can often be attributed to other pathologies, such as osteoarthritis.^1^, ^3^, ^4^, ^5^ When a meniscal tear is judged to be the cause of symptoms, surgical treatment to excise the unstable meniscal tissue—arthroscopic partial meniscectomy—is frequently recommended, and is one of the most common orthopaedic surgical procedures worldwide.^6^, ^7^ However, after the publication of the results of several randomised controlled clinical trials,^8^, ^9^, ^10^, ^11^, ^12^, ^13^ the effectiveness of arthroscopic partial meniscectomy has been debated.^14^, ^15^, ^16^, ^17^, ^18^, ^19^ In view of concerns that the procedure might be overused, a key concern is the occurrence of rare but serious complications.^20^

Estimates of the frequency of complications after arthroscopic knee surgery vary widely, and previous studies have had several limitations.^20^ In many studies, the cohorts comprised a mixture of patients who underwent major procedures with combined open techniques, such as ligament reconstruction, and those who underwent less invasive and purely arthroscopic procedures, such as arthroscopic partial meniscectomy (appendix).^20^ Studies have also frequently been limited by small population or a reliance upon regional or insurance company databases, which could under-represent the true frequency of complications. The focus of several studies was venous thromboembolic complications or infection only, and comparisons between studies are challenging because of variations in populations, age groups, health systems, insurance providers, data sources, data collection, coding, and methods. There has been increased scrutiny of the requirement for individualised patient consent to undergo surgery or another invasive procedure.^21^ Estimation of an individual's attributable risk from undergoing a procedure, rather than the unadjusted absolute risk, is crucial, and previous studies have been unable to calculate this important information because of the absence of comparison to general population data.

**Research in context**

**Evidence before this study**

A meta-analysis of studies of adverse events after knee arthroscopy, published in 2015, included summary estimates of deep vein thrombosis (0·413% [95% CI 0·178–0·960]; five studies of 432 663 patients or procedures), pulmonary embolism (0·145% [0·059–0·354]; six studies of 736 823 patients or procedures), venous thromboembolism—ie, deep vein thrombosis or pulmonary embolism—(0·568% [0·296–1·090]; six studies of 571 793 patients or procedures), infection (0·211% [0·080–0·556]; four studies of 946 230 patients or procedures), and death (0·096% [0·004–2·390; two studies of 106 967 patients or procedures). Heterogeneity (*I*^2^) in all these estimates exceeded 90% and the included studies had several limitations. Studies had inconsistent inclusion criteria, often included patients undergoing complex arthroscopic procedures (such as ligament reconstruction), and had different outcomes, endpoints, and units of analysis (ie, patient or procedure). The completeness of case capture in insurance company databases was also of concern (where applicable). Thus, CIs were wide, the range of complications reported was incomplete, and the generalisability of the findings to patients undergoing the most common procedure, arthroscopic partial meniscectomy, in isolation was unknown. We did an updated search of MEDLINE, Embase, CENTRAL, and CINAHL on Feb 8, 2018, and followed the published search strategy from the previous meta-analysis of harms. We identified four subsequent studies of venous thromboembolism and one study of infection, in which the frequencies of these complications was similar to those in the previous meta-analysis. In a cohort study of 45 943 patients, myocardial infarction occurred in 23 (0·05%) within 30 days of knee arthroscopy. For comparison, in the general population, the previously reported 90-day risk of mortality is 0·158%, of myocardial infarction is 0·058% (in men younger than 80 years; the corresponding frequency in women is 0·028%), of pulmonary embolism is 0·006%, of stroke is 0·034%, and of non-iatrogenic septic arthritis is 0·001%.

**Added value of this study**

We assessed a wide range of serious complications in a cohort restricted to patients undergoing arthroscopic partial meniscectomy only. To our knowledge, our study is the largest reported single database cohort so far of knee arthroscopy procedures (699 965 cases). Arthroscopic partial meniscectomy was associated with a 0·317% risk of serious complications within 90 days (pulmonary embolism, myocardial infarction, stroke, fasciotomy, neurovascular injury, infection requiring surgery, or death). Increasing age was associated with an increased risk of serious complications, and female patients were at decreased risk of serious complications. Compared with the general population, arthroscopic partial meniscectomy was associated with an increased risk of septic arthritis and pulmonary embolism, and neither risk has improved over time despite modern prophylactic methods. For every 1500 fewer knee arthroscopies done, one pulmonary embolism and two native knee joint infections could be prevented.

**Implications of all the available evidence**

Overall, our findings suggest that arthroscopic partial meniscectomy is a low-risk procedure, and should continue to be used in carefully selected patients. However, the increased risks of pulmonary embolism and septic arthritis, rare but serious complications, are important to consider because up to 2 million knee arthroscopies are done worldwide each year. Our data will help to inform patient decision making and consent. Continued focus on the development of refined patient selection criteria is justified to avoid exposure to potentially avoidable risks.

We aimed to comprehensively analyse the risk of complications associated with undergoing isolated arthroscopic partial meniscectomy based on the National Health Service (NHS) and Office for National Statistics (ONS) databases for England. When data were available, comparison was made to the risk of adverse events such as mortality, pulmonary embolism, myocardial infarction, and stroke in the general population, to guide patients and clinicians about the relative risk of undergoing the procedure.

## Methods

### Data sources

We did an analysis of prospectively collected, national, hospital care data in England. We obtained national Hospital Episode Statistics (HES) data from NHS Digital (application DARS-NIC-68703), which was linked with the ONS mortality dataset. HES contains a record of all patient attendances at NHS hospitals in England, and covers episodes of care delivered in treatment centres (including those in the independent sectors) funded by the NHS, episodes of care in England when patients are resident outside England, and privately funded patients treated within NHS England hospitals.^22^ The information recorded in the HES database includes patient demographic and residence data, primary and secondary diagnoses including comorbidities, and all procedures done. The ONS mortality dataset contains national death certificate data, irrespective of whether the death occurred in hospital or the community. For the purposes of this study, the date of death and cause of death (according to the 10th edition of the International Classification of Diseases and Related Health Problems [ICD-10]) were extracted and linked to the corresponding HES records for patients. Ethical approval was not required for this study.

### Procedures

We extracted data for patients who underwent arthroscopic partial meniscectomy from HES records between April 1, 1997, and March 31, 2017. Episodes were identified from the Classification of Surgical Operations and Procedures (OPCS-4) codes in the procedure fields within the HES data (W82.2).^23^ Only isolated cases of arthroscopic partial meniscectomy were included; simultaneous or staged (within 6 months) bilateral cases were excluded. Per patient, per side, only the primary arthroscopic partial meniscectomy was included as an index procedure. Subsequent revision procedures in the same patient were counted as reoperation complications if done within 90 days of the index procedure in the same knee. For each patient identified as undergoing an index arthroscopic partial meniscectomy, all the patient's previous and subsequent hospital episodes were identified for the entire data-extraction period, to identify adverse events and improve the completeness and accuracy of data extraction for each procedure and patient. Procedures for which essential data (ie, age or sex of patient, procedure date, procedure laterality) were missing were excluded from the study. Patients for whom non-essential data (ie, index of multiple deprivation, ethnicity, rurality) were missing were included in the study but excluded from analyses in which the missing variable was adjusted for.

Complications during the 90 days after the index procedure were identified by review of the 20 ICD-10 diagnosis fields per hospital episode and 24 OPCS operation fields per episode.^23^, ^24^ Complications identified from the ICD-10 diagnosis fields were pulmonary embolism, myocardial infarction, stroke, lower-respiratory-tract infection, acute kidney injury, urinary tract infection, and neurovascular injury. Complications identified from the OPCS operation codes were confirmed to match the laterality (left *vs* right) of the index procedure with the OPCS laterality codes. The procedure-based complications were joint infection, fasciotomy, and reoperation. Mortality data were extracted from the linked ONS mortality fields.

### Outcomes

The primary outcome was the occurrence of at least one serious complication within 90 days of an index arthroscopic partial meniscectomy. A serious complication was defined as either myocardial infarction, stroke, pulmonary embolism, infection requiring surgery, fasciotomy, neurovascular injury, or death. The frequency of each individual complication was then assessed secondarily, with each complication counted whether in isolation or in combination with other adverse outcomes.

### Statistical analysis

Descriptive statistics were used to report demographic data. Complication rates were reported with 95% CIs corresponding to the proportion of the study sample. In accordance with ONS and NHS Digital guidance, if fewer than six events were recorded, data were suppressed.^25^

We used logistic regression methods to first calculate the unadjusted odds of each complication by age group, sex, index of multiple deprivation (quintiles derived from regional factors in England including average income, employment, education, housing, and crime; quintile 1 includes the least deprived areas, quintile 5 includes the most deprived), ethnicity, modified Charlson co-morbidity index (Summary Hospital-level Mortality Indicator Specification; derived with maximum 5-year diagnosis code look-back period), year of treatment, ethnicity, and rurality.^26^, ^27^, ^28^, ^29^ The odds ratios (ORs) were then adjusted by including all these variables in the same model.

To guide patients and clinicians about the relative risk of undergoing arthroscopic partial meniscectomy, general population data were reviewed. Population and adverse event numbers were extracted from the ONS national mortality report, and from publications in which population-level rates of myocardial infarction, pulmonary embolus, stroke, or septic arthritis were reported.^30^, ^31^, ^32^, ^33^, ^34^ Annualised rates were adjusted directly to estimate 90-day complication rates. The relative risk of each adverse event after arthroscopic partial meniscectomy was calculated by comparing the adverse event rates in an age-matched and sex-matched sample of our study population to that calculated in the general population. The number needed to harm was calculated as the reciprocal of the risk difference between the study cohort and the general population (attributable risk). We used Stata (version 15.1) for all analyses.

### Role of the funding source

The study sponsors had no role in study design; data collection, analysis, or interpretation; or writing of the report. All authors had access to all study data and had final responsibility for the decision to submit for publication.

## Results

**Figure 1:**
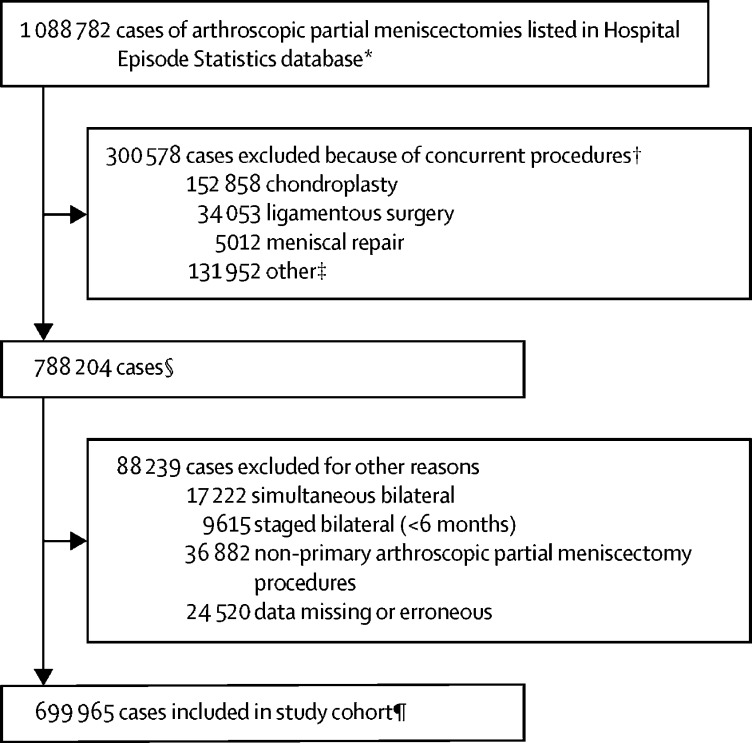
Case selection *938 612 patients. †Same side or contralateral surgical procedures (not mutually exclusive). ‡Includes primary or revision total knee arthroplasty, unspecified meniscal surgery, total meniscectomy, meniscal transplant, fusion, interposition, washout, synovial or fat pad procedures. §702 401 patients. ¶666 442 patients.

Between April 1, 1997, and March 31, 2017, 1 088 782 arthroscopic partial meniscectomies were done. 699 965 isolated procedures in 666 442 patients were eligible for inclusion as index procedures (figure 1). In table 1 we summarise the characteristics of the overall cohort and patients developing serious complications within 90 days. Arthroscopic partial meniscectomies were done more frequently in men (453 726 [64·82%] cases) than women (246 239 [35·18%] cases) and most commonly in people aged 40–59 years (330 752 [47·25%] cases; table 1).

**Table 1:** Demographics and descriptive statistics

		**All procedures**	Serious complications*
Overall		699 965 (100%)	2218 (0·32%, 0·30–0·33)
Sex			
	Male	453 726 (64·82%)	1545 (0·34%, 0·32–0·36)
	Female	246 239 (35·18%)	673 (0·27%, 0·25–0·29)
Age group, years			
	<20	21 798 (3·11%)	32 (0·15%, 0·10–0·21)
	20–39	172 636 (24·66%)	356 (0·21%, 0·19–0·23)
	40–59	330 752 (47·25%)	988 (0·30%, 0·28–0·32)
	60–79	167 287 (23·90%)	766 (0·46%, 0·43–0·49)
	≥80	7492 (1·07%)	76 (1·01%, 0·80–1·27)
Modified Charlson comorbidity index			
	0	590 110 (84·31%)	1660 (0·28%, 0·26–0·30)
	1–15	105 689 (15·10%)	497 (0·47%, 0·43–0·51)
	16–30	3881 (0·55%)	52 (1·34%, 1·00–1·75)
	31–50	285 (0·04%)	9 (3·16%, 1·45–5·91)
Index of multiple deprivation			
	Quintile 1	168 715 (24·10%)	489 (0·29%, 0·26–0·32)
	Quintile 2	156 657 (22·38%)	493 (0·31%, 0·29–0·34)
	Quintile 3	145 000 (20·72%)	497 (0·34%, 0·31–0·37)
	Quintile 4	123 913 (17·70%)	403 (0·33%, 0·29–0·36)
	Quintile 5	100 320 (14·33%)	326 (0·32%, 0·29–0·36)
	Missing	5360 (0·77%)	..
Rurality			
	Urban	547 459 (78·21%)	1712 (0·31%, 0·30–0·33)
	Rural	149 882 (21·41%)	504 (0·34%, 0·31–0·37)
	Missing	2624 (0·37%)	..
Ethnic origin			
	White	564 320 (80·62%)	1911 (0·34%, 0·32–0·35)
	Asian	18 555 (2·65%)	51 (0·27%, 0·20–0·36)
	Black	11 222 (1·60%)	41 (0·37%, 0·26–0·50)
	Mixed race	3870 (0·55%)	13 (0·34%, 0·18–0·57)
	Other	6547 (0·94%)	14 (0·21%, 0·12–0·36)
	Missing	95 451 (13·64%)	..

Data are n (%) or n (%, 95% CI)

* At least one serious complication within 90 days, defined as either pulmonary embolism, myocardial infarction, stroke, infection requiring surgery, fasciotomy, neurovascular injury, or death.

4239 cases of reoperation (planned or unplanned) were recorded (0·606% [95% CI 0·588–0·624]; table 2). Of these reoperations, 944 (0·135% [0·126–0·144]) were done because of infection (table 2). Serious complications occurred in 2218 cases (0·317% [0·304–0·330]), and 217 deaths were recorded within 90 days of the index procedure (0·031% [0·027–0·035]). Pulmonary embolism occurred in 546 cases (0·078% [0·072–0·085]), ten of which were fatal (0.001% [0·000–0·003]; table 2).

**Table 2:** Complications within 90 days

	**n (% [95% CI])**
Any reoperation*	4239 (0·606% [0·588–0·624])
Serious complication†	2218 (0·317% [0·304–0·330])
Infection‡	944 (0·135% [0·126–0·144])
Lower-respiratory-tract infection	931 (0·133% [0·125–0·142])
Urinary tract infection	647 (0·092% [0·085–0·100])
Pulmonary embolism	546 (0·078% [0·072–0·085])
Myocardial infarction	279 (0·040% [0·035–0·045])
Mortality	217 (0·031% [0·027–0·035])
Stroke	208 (0·030% [0·026–0·034])
Acute kidney injury	206 (0·029% [0·026–0·034])
Neurovascular injury	67 (0·010% [0·007–0·012])
Fasciotomy	33 (0·005% [0·003–0·007])
Fatal pulmonary embolism	10 (0·001% [0·000–0·003])

N=699 965.

* Any procedure done in the same knee (eg, washout, meniscal repair, repeat meniscectomy, chondroplasty, ligamentous surgery, fasciotomy).

† At least one serious complication within 90 days, defined as either pulmonary embolism, myocardial infarction, stroke, infection requiring surgery, fasciotomy, neurovascular injury, or death.

‡ Infection requiring surgery (open or arthroscopic washout).

**Figure 2:**
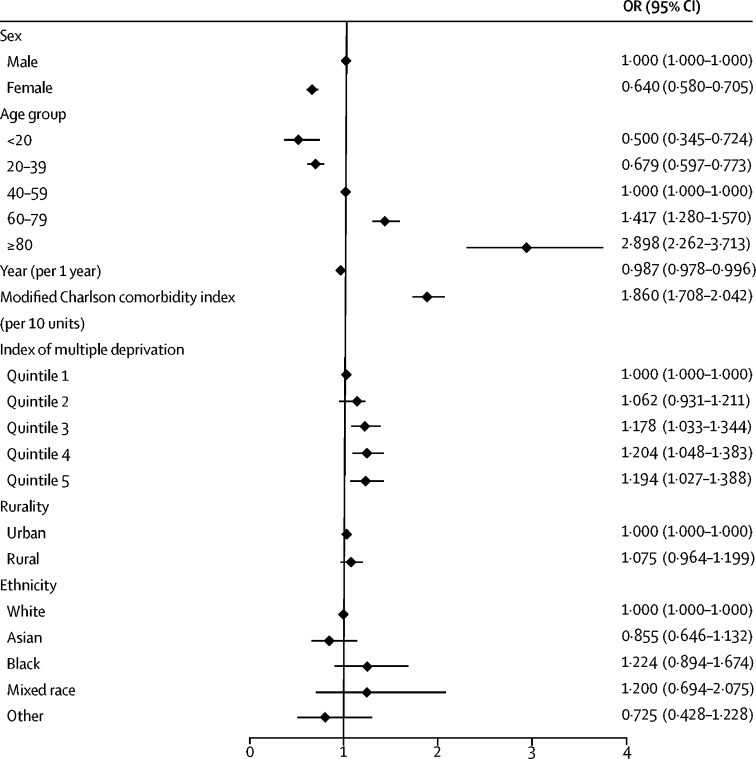
Forest plot of adjusted odds of any serious complication within 90 days Procedure-level multivariable logistic regression model adjusted for sex, age group, year, modified Charlson comorbidity index, index of multiple deprivation, rurality, and ethnicity. Error bars show 95% CIs. OR=odds ratio.

Female sex was associated with a decreased risk of complications compared with male sex (OR 0·640 [95% CI 0·580–0·705]; figure 2), partly driven by an increased risk of myocardial infarction and infection in male patients (table 3). Increasing age was associated with an increased risk of serious complications (adjusted OR 1·247 per decade [95% CI 1·208–1·288]). Serious complications occurred in 76 (1·01%) cases in patients aged 80 years or older and 32 (0·15%) of cases in patients younger than 20 years, a difference that corresponded to an adjusted OR of 5·794 (95% CI 3·740–8·975). Increased comorbidity was associated with an increased risk of myocardial infarction, stroke, infection, death, and the overall risk of serious complications (table 3). Complications occurred more frequently in areas of increased deprivation (figure 2; table 3). Rurality and ethnicity did not affect the risk of serious complications (figure 2; table 3). The rate of serious complications fell slightly during the 20-year study period (adjusted OR 0·987 per year [95% CI 0·978–0·996]; table 3).

**Table 3:** Unadjusted and adjusted odds of serious complications

	Serious complication*		**Pulmonary embolism**		**Myocardial infarction**		**Stroke**		**Infection**†		**Death**	
	Crude OR (95% CI)	Adjusted OR (95% CI)	Crude OR (95% CI)	Adjusted OR (95% CI)	Crude OR (95% CI)	Adjusted OR (95% CI)	Crude OR (95% CI)	Adjusted OR (95% CI)	Crude OR (95% CI)	Adjusted OR (95% CI)	Crude OR (95% CI)	Adjusted OR (95% CI)
**Sex**												
Male	1·00	1·00	1·00	1·00	1·00	1·00	1·00	1·00	1·00	1·00	1·00	1·00
Female	0·802 (0·733–0·878)	0·640 (0·580–0·705)	1·311 (1·106–1·555)	1·021 (0·848–1·228)	0·571 (0·433–0·752)	0·406 (0·303–0·543)	1·325 (1·006–1·745)	0·818 (0·611–1·096)	0·492 (0·421–0·575)	0·454 (0·385–0·536)	1·210 (0·922–1·588)	0·852 (0·635–1·143)
**Age group, years**												
<20	0·491 (0·345–0·698)	0·500 (0·345–0·724)	..	..	..	..	..	..	0·993 (0·677–1·455)	0·925 (0·617–1·387)	0·214 (0·030–1·538)	0·261 (0·036–1·883)
20–39	0·690 (0·611–0·779)	0·679 (0·597–0·773)	0·482 (0·369–0·631)	0·481 (0·358–0·646)	0·029 (0·007–0·116)	0·015 (0·002–0·109)	0·133 (0·054–0·329)	0·157 (0·063–0·392)	1·119 (0·957–1·308)	1·051 (0·891–1·239)	0·432 (0·251–0·743)	0·476 (0·269–0·841)
40–59	1·00	1·00	1·00	1·00	1·00	1·00	1·00	1·00	1·00	1·00	1·00	1·00
60–79	1·535 (1·397–1·688)	1·417 (1·280–1·570)	1·524 (1·270–1·829)	1·422 (1·166–1·734)	1·933 (1·518–2·462)	1·794 (1·377–2·338)	3·077 (2·288–4·137)	2·425 (1·761–3·340)	1·053 (0·897–1·237)	1·081 (0·911–1·282)	2·842 (2·099–3·847)	2·533 (1·814–3·536)
≥80	3·420 (2·706–4·324)	2·898 (2·262–3·713)	1·328 (0·657–2·684)	1·122 (0·525–2·399)	4·654 (2·682–8·076)	4·065 (2·277–7·257)	11·677 (7·040–19·369)	6·941 (4·017–11·993)	1·032 (0·551–1·932)	1·119 (0·593–2·112)	16·846 (10·809–26·254)	11·620 (7·044–19·169)
**Year of treatment (per year)**												
Year	1·000 (0·992-1·008)	0·987 (0·978–0·996)	1·010 (0·994–1·027)	0·997 (0·979–1·016)	1·012 (0·989–1·035)	0·983 (0·958–1·009)	1·025 (0·998–1·053)	0·992 (0·962–1·023)	0·992 (0·980–1·004)	0·989 (0·976–1·002)	0·990 (0·965–1·016)	0·965 (0·937–0·994)
**Modified Charlson comorbidity index (per unit)**												
Charlson index	1·083 (1·074–1·091)	1·064 (1·055–1·074)	1·048 (1·026–1·071)	1·023 (0·998–1·048)	1·113 (1·095–1·131)	1·081 (1·061–1·102)	1·144 (1·129–1·160)	1·110 (1·092–1·128)	1·027 (1·007–1·047)	1·030 (1·009–1·050)	1·133 (1·116–1·150)	1·099 (1·079–1·119)
**Index of multiple deprivation (quintile)**												
1	1·00	1·00	1·00	1·00	1·00	1·00	1·00	1·00	1·00	1·00	1·00	1·00
2	1·086 (0·958–1·231)	1·062 (0·931–1·211)	1·016 (0·788–1·309)	0·924 (0·702–1·218)	1·239 (0·876–1·751)	1·224 (0·850–1·761)	1·180 (0·776–1·792)	1·155 (0·748–1·783)	1·108 (0·916–1·341)	1·106 (0·908–1·346)	1·051 (0·680–1·624)	1·131 (0·718–1·781)
3	1·183 (1·044–1·341)	1·178 (1·033–1·344)	1·211 (0·945–1·551)	1·249 (0·961–1·624)	1·144 (0·799–1·639)	1·134 (0·774–1·661)	1·413 (0·939–2·126)	1·460 (0·958–2·224)	1·153 (0·951–1·397)	1·132 (0·928–1·382)	1·362 (0·898–2·067)	1·330 (0·849–2·082)
4	1·123 (0·984–1·281)	1·204 (1·048–1·383)	1·052 (0·805–1·375)	1·243 (0·939–1·646)	0·976 (0·660–1·444)	1·077 (0·709–1·636)	1·232 (0·794–1·911)	1·378 (0·873–2·177)	1·140 (0·933–1·394)	1·131 (0·917–1·396)	1·727 (1·147–2·601)	2·030 (1·310–3·145)
5	1·122 (0·975–1·291)	1·194 (1·027–1·388)	1·108 (0·837–1·466)	1·289 (0·952–1·744)	1·318 (0·899–1·930)	1·684 (1·123–2·526)	1·241 (0·780–1·975)	1·323 (0·800–2·186)	1·063 (0·854–1·321)	1·009 (0·800–1·273)	1·477 (0·944–2·311)	1·806 (1·107–2·944)
**Rurality**												
Urban	1·00	1·00	1·00	1·00	1·00	1·00	1·00	1·00	1·00	1·00	1·00	1·00
Rural	1·076 (0·974–1·188)	1·075 (0·964–1·199)	1·121 (0·920–1·367)	1·059 (0·849–1·323)	1·247 (0·952–1·633)	1·144 (0·846–1·548)	1·218 (0·890–1·667)	1·103 (0·780–1·558)	1·008 (0·863–1·177)	1·058 (0·895–1·250)	1·094 (0·797–1·500)	1·285 (0·908–1·816)
**Ethnicity**												
White	1·00	1·00	1·00	1·00	1·00	1·00	1·00	1·00	1·00	1·00	1·00	1·00
Asian	0·811 (0·614–1·072)	0·855 (0·646–1·132)	0·388 (0·173–0·868)	0·407 (0·182–0·914)	1·273 (0·676–2·396)	1·490 (0·786–2·824)	0·835 (0·344–2·032)	0·964 (0·394–2·360)	0·924 (0·620–1·376)	0·929 (0·622–1·388)	0·840 (0·345–2·043)	0·967 (0·395–2·369)
Black	1·079 (0·792–1·471)	1·224 (0·894–1·674)	0·642 (0·287–1·436)	0·676 (0·300–1·520)	0·210 (0·030–1·499)	0·278 (0·039–1·990)	1·105 (0·410–2·977)	1·481 (0·541–4·055)	1·590 (1·076–2·350)	1·725 (1·160–2·565)	1·111 (0·413–2·994)	1·375 (0·504–3·751)
Mixed race	0·992 (0·574–1·713)	1·200 (0·694–2·075)	0·620 (0·155–2·488)	0·767 (0·191–3·083)	0·610 (0·086–4·349)	1·031 (0·144–7·374)	0·801 (0·112–5·720)	1·386 (0·193–9·931)	1·596 (0·827–3·081)	1·638 (0·846–3·171)	..	..
Other	0·631 (0·373– 1·068)	0·725 (0·428–1·228)	0·550 (0·177–1·712)	0·633 (0·203–1·975)	0·361 (0·051–2·570)	0·531 (0·074–3·792)	0·474 (0·066–3·380)	0·761 (0·106–5·449)	0·419 (0·157–1·118)	0·419 (0·157–1·121)	1·905 (0·707–5·133)	2·756 (1·017–7·471)

Procedure-level multivariable logistic regression model adjusted for sex, age group, year, modified Charlson comorbidity index, index of multiple deprivation, rurality, and ethnicity. OR=odds ratio.

* At least one serious complication within 90 days, defined as either pulmonary embolism, myocardial infarction, stroke, infection requiring surgery, fasciotomy, neurovascular injury, or death.

† Infection requiring surgery (open or arthroscopic washout).

The risk of 90-day mortality was lower in the study population undergoing arthroscopic partial meniscectomy than the national rate of mortality (excluding deaths from cancer) in patients aged 40 years or older (table 4). The risk of myocardial infarction and stroke in our cohort was similar to or lower than that in the general population (table 4). The relative risk of pulmonary embolism (risk ratio 12·99 [95% CI 10·35–16·31]) and septic arthritis (110·21 [81·19–149·60]) were higher in our study population than in the general population. For pulmonary embolism, the estimated number needed to harm was 1390 (95% CI 1272–1532). For native knee joint infection, the estimated number needed to harm was 749 (95% CI 704–801). We noted ten (1·8%) fatal pulmonary embolisms among the 546 cases recorded, and estimated one attributable death from pulmonary embolism for every 77 519 arthroscopic partial meniscectomies (95% CI 45 455–261 097).

**Table 4:** 90-day adverse event rates in the general population *vs* a matched sample of the study cohort

		**General population risk % (95% CI)**	**Study cohort risk % (95% CI)**	**Risk ratio (95% CI)**
Mortality^30^*				
	Overall	0·158% (0·157–0·159)	0·031% (0·027–0·035)	0·20 (0·17–0·22)
	<20 years	0·007% (0·007–0·008)	..	..
	20–39 years	0·011% (0·011–0·012)	0·009% (0·005–0·015)	0·81 (0·49–1·32)
	40–59 years	0·043% (0·042–0·044)	0·021% (0·017–0·027)	0·50 (0·40–0·63)
	60–79 years	0·233% (0·230–0·236)	0·061% (0·050–0·074)	0·26 (0·22–0·32)
	≥80 years	2·043% (2·026–2·060)	0·360% (0·238–0·524)	0·18 (0·12–0·26)
Myocardial infarction^31^				
	Men (<80 years)	0·058% (0·049–0·067)	0·046% (0·040–0·052)	0·79 (0·64–0·97)
	Women (<80 years)	0·028% (0·022–0·035)	0·024% (0·019–0·031)	0·88 (0·63–1·23)
Pulmonary embolism^32^		0·006% (0·005–0·007)	0·078% (0·072–0·085)	12·99 (10·35–16·31)
Stroke (<75 years)^33^		0·034% (0·030–0·039)	0·025% (0·021–0·029)	0·72 (0·59–0·88)
Septic arthritis (native knee joint infection)^34^		0·001% (0·001–0·002)†	0·135% (0·126–0·144)	110·21 (81·19–149·60)

* Mortality data are Office for National Statistics population-level data for England, 2016, excluding death from cancer.

† Excludes iatrogenic causes.

## Discussion

Our study of 699 965 cases of arthroscopic partial meniscectomy shows that complications occur rarely after the procedure. Although the risk of some complications increased with increasing age, in patients with comorbidities, and in men, most adverse events were less common in our study population than in the general population (table 4). We noted a small but significant decrease in the risk of mortality after arthroscopic partial meniscectomy during the 20-year study period, which has been previously reported for total joint arthroplasty surgery.^35^, ^36^ The risk of infection and pulmonary embolism, however, was substantially increased after arthroscopic partial meniscectomy, with a number needed to harm of 1390 for pulmonary embolism and 749 for native knee joint infection. One death from pulmonary embolism could be prevented if 77 519 fewer arthroscopic partial meniscectomies were done.

Reports of several clinical trials of arthroscopic partial meniscectomy have now been published and their findings suggest that, for many patient groups, exercise therapy could be an effective treatment.^8^, ^9^, ^10^, ^11^, ^12^, ^13^ In the trials published so far, patients had so-called degenerative meniscal tears and mean age has ranged from 47 to 58.^8^, ^9^, ^10^, ^11^, ^12^, ^13^ The trial evidence applicable to this population can now be interpreted in the context of risk, and our study provides an indication of the potential morbidity associated with overuse of knee arthroscopy.

The absolute rate of pulmonary embolism, infection, and death in our study was less than the pooled estimate reported by a meta-analysis^20^ of previous studies (appendix). This finding is likely to be related to reduced heterogeneity in our analysis, which was limited to patients undergoing isolated arthroscopic partial meniscectomy and excluded complex procedures such as anterior cruciate ligament reconstruction. Nevertheless, we estimated a 13-times greater risk of developing a pulmonary embolism after the procedure compared with the risk in the general population (table 4), which probably reflects the fact that only around 25% of pulmonary embolisms occur in unprovoked circumstances (ie, without a history of surgery, cancer, hospitalisation, pregnancy, or trauma).^32^, ^37^ The risk of pulmonary embolism did not change in our cohort over time (table 3), despite modern attention to prevention of venous thromboembolism.^38^ The rate of fatal pulmonary embolism in our series was similar to that in other series.^39^ Although rare, the increased risk of pulmonary embolism is concerning because thromboprophylaxis is not advocated routinely for people undergoing knee arthroscopy.^40^ As shown in studies^35^, ^36^ of patients undergoing joint arthroplasty, venous thromboembolism can be prevented with mechanical and chemical prophylaxis, but any potential benefits must be weighed against the increased risk of bleeding. Our findings could inform a further review of thromboprophylaxis recommendations for patients undergoing arthroscopic partial meniscectomy, especially those in high-risk groups.

The overall risk of undergoing further surgery for infection within 90 days was also increased compared with the general population (table 4). Male patients and patients with comorbidities had an increased risk of undergoing surgery for infection (table 3). Whether antibiotic prophylaxis should be routinely given before knee arthroscopy is debated.^41^, ^42^ The results of a study^43^ published in 1988 suggested that prophylactic antibiotics might be cost effective if the infection rate associated with knee arthroscopy were greater than 0·08%. The rate of reoperation because of infection was higher than this threshold in our cohort. However, whether patients received antibiotic prophylaxis at the time of their index procedure was unknown. A contemporary cost-effectiveness analysis is warranted to support any recommendations about routine prophylaxis.

For low-risk procedures, the rate of complications compared with that in the general population is important to consider. This comparison is important to inform patients and clinicians of the relative risk of undergoing arthroscopic partial meniscectomy.^21^ For example, the risk of myocardial infarction in our cohort was similar in women to, and lower in men than, that reported in the general population, in whom the 90-day incidence in people younger than 80 years is around 0·058% in men and 0·028% in women.^31^ Similarly, stroke risk in our cohort was slightly lower than that in the general population younger than 75 years.^33^ Arthroscopic partial meniscectomy was most commonly done in patients aged 40–59 years, and 90-day mortality in this age group was 0·021%, compared with 0·043% in the general population (excluding deaths from cancer).^30^ Although this comparison is crude, the difference is similar to the 39–43% relative mortality reported in patients undergoing total knee or total hip arthroplasty compared with the general population.^44^ The specific cause of the decreased mortality in our cohort relative to the general population is unknown, but is probably a so-called healthy patient selection bias, which is affected by differences in health-care seeking behaviour and access to surgical treatment.^45^ The causes of the decreased mortality are likely to be many, and in the UK, for example, decreased mortality has been reported in individuals with specific occupations.^46^ Patients' willingness to seek medical attention could be affected by the relative severity of a coexisting condition or by socioeconomic status. Equally, surgeons or anaesthetists might recommend surgery less readily in patients whom they judge to be high risk because of the presence of comorbid disorders. In a study^44^ of patients who underwent hip or knee arthroplasty, a reduced frequency of death from cancer was a key driver for reduced mortality in these patients relative to the general population.^30^ In our cohort, mortality was decreased even after deaths from cancer were excluded from the national population data. Increased mortality in the general population could still have been driven, for example, by over-representation of patients with severe cardiac or respiratory comorbidities. The risk of pulmonary embolism and infection only were greater in our cohort than in the general population. However, no comparison group was available for rarer direct surgical complications (eg, neurovascular injury, fasciotomy).

One of the main criticisms of previous studies of the risks of knee arthroscopy has been the analysis of cohorts comprising both patients undergoing simple, isolated, procedures such as arthroscopic partial meniscectomy and those undergoing complex and multiple procedures. No previous study has focused solely on arthroscopic partial meniscectomy. To reduce heterogeneity, we carefully limited our analysis to isolated cases, and excluded patients undergoing bilateral surgery or any concurrent procedures. Application of these strict criteria meant that only 699 965 (64%) of 1 088 782 procedures in the HES database were included, yet our study remains the largest arthroscopic partial meniscectomy cohort reported so far. Our large sample of prospectively recorded data enabled us to carefully control against potential confounding factors and increased the precision of our findings. Patient factors (especially age group, sex, and comorbidities) are probably more important than the health-care delivery setting in estimation of the risk of adverse events, and thus our findings are likely to be broadly generalisable to other health-care settings and countries. Potential sources of unmeasured confounding, however, include body-mass index, smoking status, and issues of data quality, such as incomplete coding of comorbidity, indication, or procedure. Furthermore our findings cannot be generalised to patients undergoing more complex arthroscopic surgery.

We analysed observational HES data, as recorded by hospitals in England for the purposes of reimbursement for treatment, clinical audit, and research. A wealth of data is recorded, but data quality varies between recorded fields. As hospitals rely on the coding of surgical procedures and serious acute medical diagnoses for financial reimbursement, there is a strong incentive to correctly and reliably enter these data, which we used in our study. In support of this reasoning, the modified Charlson comorbidity index as calculated from HES diagnosis fields and records of serious vascular complications correlate strongly with primary care records.^47^, ^48^ Other fields are less reliably coded. Ethnicity data, for example, was missing for 13·64% of cases in our cohort. To minimise the effect of missing data, look-back and look-forward analyses of other hospital episodes for the same patient were done when appropriate but, without direct validation, missing or inaccurately coded data remains a possibility. Although a limitation of the HES database is that private hospital treatment is not recorded, we believe that the effect of this omission on our findings will have been minimal. Patients undergoing a primary procedure in the NHS are highly likely to have returned to an NHS hospital after development of an adverse event. Because the HES records hospital attendances across England, complications within 90 days are unlikely to have been missed because of migration.

Our study included death certificate data from the ONS, which maintains a complete national record of both in-hospital and community deaths. Our analysis was, however, restricted to hospital episodes, and complications managed in the community were not available. For example, because a large proportion of patients with symptomatic deep vein thromboses are now diagnosed and treated in the community, this complication was not included as an outcome in our study. Any analysis of the frequency of deep vein thrombosis based on hospital episodes only would probably underestimate the true rate. The purpose of our study was to establish the rate of serious complications diagnosed in hospital. We comprehensively report the full range of the serious complications that might occur, rather than focusing on a few specific complications, as has been done in previous studies.

Clinical trial evidence, published mainly in the past 6 years, has challenged the efficacy of arthroscopic partial meniscectomy, and the procedure is now advocated only in patients with specific radiological findings that correspond with symptoms, and only after a trial of conservative treatment. Overall, our study suggests that arthroscopic partial meniscectomy is a low-risk procedure, with no detected attributable risk of myocardial infarction, stroke, or death. Our findings support potential use of arthroscopic partial meniscectomy in carefully selected patients. When the anticipated benefit is low, however, overuse of the procedure is a concern and the potential morbidity from rare but serious complications should be considered. Specifically, we estimated that one pulmonary embolism could be avoided for every 1390 fewer arthroscopic partial meniscectomies done, and one native knee joint infection could be avoided for every 749 fewer procedures done. The risks of pulmonary embolism and infection have not changed over time despite modern approaches to prevention. In view of the current scrutiny of shared decision making and individualised consent, our findings will help clinicians and patients to balance anticipated benefits and risks. The focus on implementation of enhanced treatment guidelines is justified to reduce avoidable risk and morbidity.

## Data sharing
