# Supplementary material for: Adverse outcomes after arthroscopic partial meniscectomy: a study of 700 000 procedures in the national Hospital Episode Statistics database for England
Source: Lancet. 2018 Nov 17;392(10160):2194–202. doi: 10.1016/S0140-6736(18)31771-9 (PMC6238020; doi:10.1016/S0140-6736(18)31771-9)
Supplement: Supplementary appendix [file mmc1.pdf]

THE LANCET

Supplementary appendix

This appendix formed part of the original submission and has been peer reviewed. We post it as supplied by the authors.

Supplement to: Abram SGF, Judge A, Beard DJ, Price AJ. Adverse outcomes after arthroscopic partial meniscectomy: a study of 700 000 procedures in the national Hospital Episode Statistics database for England. *Lancet* 2018; published online September 24. http://dx.doi.org/10.1016/S0140-6736(18)31269-8.

Appendix: Previous studies of knee arthroscopy risks

**Reference**	**Cohort (country)**	**Open procedures (e.g. ACLr) excluded?**	**Procedures n**	**Period (days)**	**DVT n (%)**	**PE n (%)**	**VTE n (%)**	**MI n (%)**	**CVA n (%)**	**Reoperation n (%)**	**Infection n (%)**	**Death n (%)**
Murphy 2016[1]	Single centre (USA)	No	2,783	30			7 (0.25%)					
Shah 2017[2]	National database (USA)	Unclear	45,943	30				- (0.05%)				
Cancienne 2017[3]	Insurer database (USA)	Unclear	1. 100,399<br>2. 629,841	90							1. 250 (0.25%)<br>2. 1755 (0.28%)	
Maletis 2012[4]	Insurer database (USA)	No	20,770	90	51 (0.25%)	35 (0.17%)	84 (0.40%)	-	-	-	-	9 (0.04%)
Hetsroni 2011[5]	Regional database (USA)	No	418,323	90	-	117 (0.03%)	-	-	-	-	-	-
Yeranosian 2013[6]	Insurer database (USA)	No	432,038	30	-	-	-	-	-	-	638 (0.15%)	-
Bohensky 2014[7]	Regional database (Australia)	Yes	166,770	30	444 (0.27%)	129 (0.08%)	573 (0.34%)	-	-	305 (0.18%)	141 (0.08%)	23 (0.01%)
Hame 2012[8]	Insurer database (USA)	No	314,578 (age >65)	90	2507 (0.80%)	982 (0.31%)		-	-	-	1107 (0.35%)	-
Jameson 2011[9]	National database (UK)	No	301,701	30/90	369 (0.12%)	230 (0.08%)	580 (0.19%)	48 (0.02%)	52 (0.02%)	1033 (0.34%)	-	47 (0.02%)

*ACLr = anterior cruciate ligament reconstruction; * - = not reported.*

References:

1 Murphy RF, Heyworth B, Kramer D, *et al.* Symptomatic Venous Thromboembolism After Adolescent Knee Arthroscopy. *J Pediatr Orthop* 2016; **0**: 1–5.

2 Shah CK, Keswani A, Boodaie BD, Yao DH, Koenig KM, Moucha CS. Myocardial Infarction Risk in Arthroplasty vs Arthroscopy: How Much Does Procedure Type Matter? *J Arthroplasty* 2017; **32**: 246–51.

3 Cancienne JM, Mahon HS, Dempsey IJ, Miller MD, Werner BC. Patient-related risk factors for infection following knee arthroscopy: An analysis of over 700,000

patients from two large databases. *Knee* 2017; **24**: 594–600.

4 Maletis GB, Inacio MC, Reynolds S, Funahashi TT. Incidence of Symptomatic Venous Thromboembolism After Elective Knee Arthroscopy. *J Bone Jt Surgery-American Vol* 2012; **94**: 714–20.

5 Hetsroni I, Lyman S, Do H, Mann G, Marx RG. Symptomatic pulmonary embolism after outpatient arthroscopic procedures of the knee: the incidence and risk factors in 418,323 arthroscopies. *J Bone Joint Surg Br* 2011; **93**: 47–51.

6 Yeranosian MG, Petrigliano FA, Terrell RD, Wang JC, McAllister DR. Incidence of postoperative infections requiring reoperation after arthroscopic knee surgery. *Arthrosc - J Arthrosc Relat Surg* 2013; **29**: 1355–61.

7 Bohensky MA, Ademi Z, DeSteiger R, *et al.* Quantifying the excess cost and resource utilisation for patients with complications associated with elective knee arthroscopy: A retrospective cohort study. *Knee* 2014; **21**: 491–6.

8 Hame SL, Nguyen V, Ellerman J, Ngo SS, Wang JC, Gamradt SC. Complications of arthroscopic meniscectomy in the older population. *Am J Sports Med* 2012; **40**: 1402–5.

9 Jameson SS, Dowen D, James P, *et al.* The burden of arthroscopy of the knee: a contemporary analysis of data from the English NHS. *J Bone Joint Surg Br* 2011; **93**: 1327–33.
